# The Efficacy of Percutaneous Transhepatic Gallbladder Drainage on Acute Cholecystitis in High-Risk Elderly Patients Based on the Tokyo Guidelines: A Retrospective Case–Control Study

**DOI:** 10.1097/MD.0000000000001442

**Published:** 2015-08-28

**Authors:** Qingqiang Ni, Dongbo Chen, Rui Xu, Dong Shang

**Affiliations:** From the Medical college of Soochow University, Suzhou, Jiangsu (QN); Department of General Surgery, Pancreato-Biliary Center, First Affiliated Hospital, Dalian Medical University, Dalian, Liaoning (QN, DC, DS); Department of General Surgery, Fujian Provincial Longyan First Hospital (DC) and Department of Cardiology, Shandong Provincial Qianfoshan Hospital, Shandong University, Jinan, Shandong, P.R. China (RX).

## Abstract

To evaluate the efficacy of percutaneous transhepatic gallbladder drainage (PTGD) for high-risk elderly patients with acute cholecystitis.

Retrospective analysis of 159 acute cholecystitis patients who were admitted to General Surgery Division III of the First Affiliated Hospital of Dalian Medical University between January 2005 and November 2012. A total of 123 patients underwent laparoscopic cholecystectomy (LC), and 36 received only PTGD treatment. The LC patients were divided into 3 groups based on their preoperative treatment: group A, emergency patients (33 patients); group B (26 patients), patients who were treated with PTGD prior to LC; and group C (64 patients), patients who received nonsurgical treatment prior to LC. General conditions, LC surgery duration, intraoperative blood loss, rate of conversion to open surgery, incidence of postoperative complications, total fasting time, and total hospitalization time were analyzed and compared among the 3 groups.

The remission rates of patients in the PTGD treatment groups (including group B and PTGD treatment only group) were significantly higher within 24 and 48 hours than those of patients who received nonsurgical treatment prior to LC (*P* < 0.05). Among the patients in the 3 surgery groups, the operation conversion rate (19.2%) of group B was significantly higher than that of group A (3.0%) and group C (1.6%) (*P* < 0.05). The total hospitalization time of the patients in group B (18.5 ± 4.5 days) was longer than that of the patients in group A (8.2 ± 3.9 days) and group C (10.5 ± 6.4 days). The total fasting time of the patients in group A (2.4 ± 1.2 days) was significantly shorter than that of those in group B (4.1 ± 1.7 days) and group C (3.4 ± 2.7 days) (*P* < 0.05).

For high-risk elderly patients, if there is any emergency surgery contraindication, PTGD therapy may be safe and effective and can relieve the symptoms within a short time. For acute cholecystitis patients without surgery contraindications, emergency surgery should be performed as soon as possible after diagnosis.

## INTRODUCTION

Acute cholecystitis is a common disease for which the best treatment approach is surgical removal of the gallbladder. In the past 20 years, laparoscopic cholecystectomy (LC) has basically replaced the traditional open cholecystectomy (OC), and LC is currently a standard treatment for acute and chronic cholecystitis. For most acute cholecystitis patients, symptoms and signs are soon alleviated after nonsurgical treatment, whereas emergency surgery is associated with increased risk of postoperative complications and mortality.

A study of more than 2000 patients by Giger et al^1^ showed that emergency surgery is a risk factor for perioperative complications and that nonsurgical treatment for a certain time period limits local inflammation. Therefore, for such patients, finding a time to perform LC surgery 48 to 72 hours after nonsurgical treatment is a better method.^2^ For high-risk acute cholecystitis patients complicated with other medical illness, both emergency surgery and surgery at a selected time after nonsurgical treatment are associated with higher postoperative complication rate and mortality. Percutaneous transhepatic gallbladder drainage (PTGD), a technique developed by Radder in 1980,^3^ has gradually become an emergency replacement therapy for high-risk acute cholecystitis patients. It has the advantages of causing little injury, relatively simple operation and lower complication rate, and it has been widely used clinically. Although these clinically common treatments have been accepted by physicians, many surgeons are still reluctant to choose PTGD for the treatment of acute cholecystitis. One of the important reasons for this is that the indications for the use of each strategy are not clear.

The present article discusses suitable strategies for LC for acute cholecystitis patients by comparing the efficacy of 3 treatment strategies: emergency LC, LC after nonsurgical treatment, and LC after PTGD treatment. We also compared postoperative complication rate, total hospitalization time, and total fasting time. In addition, by conducting follow-up on patients who only received PTGD treatment, we evaluated the feasibility of PTGD as initial treatment for high-risk elderly acute cholecystitis patients.

## MATERIALS AND METHODS

### General Information

Written informed consents were obtained from the patient for publication of this case–control study. This study was approved by the Ethics Committee of the First Affiliated Hospital of Dalian Medical University (Dalian, Liaoning, China). We performed a retrospective analysis of 159 acute cholecystitis patients who were admitted to General Surgery Division III of the First Affiliated Hospital of Dalian Medical University between January 2005 and November 2012. Of the patients, 84 were males and 75 were females. The ratio of males to female was 1.12:1. The ages of the patients were 15 to 90 years, and the average age was 62.7 ± 14.3 years. Thirty three patients underwent emergency LC, whereas 64 patients first received nonsurgical treatment before an LC was performed at a selected time. Sixty two patients underwent emergency PTGD treatment; 26 (41.9%) of these patients later returned to the hospital for LC, while the other 36 patients were discharged with tubes and did not return for cholecystectomy. Patients who underwent LC were divided into 3 groups based on preoperative treatment: group A, emergency patients who underwent LC; group B, patients who were treated with PTGD prior to LC; and group C, patients who received nonsurgical treatment prior to LC. We performed telephone follow-up on the patients who had only undergone PTGD. We discuss the efficacy of PTGD and its feasibility as initial treatment for acute cholecystitis.

### Diagnosis and Grading Criteria

#### Diagnostic Criteria for Acute Cholecystitis^4^

1. Local inflammatory signs: Murphy sign is positive; right upper quadrant mass/pain/tenderness;
2. Systemic inflammatory signs: fever, increased CRP level, and elevated white blood cell count; and
3. Imaging signs: imaging suggests acute cholecystitis.

Ultrasound: Ultrasound Murphy sign is positive (pressing gallbladder with an ultrasound probe reveals tenderness); gallbladder wall thickening (>4 mm, with exceptions for chronic liver disease, ascites, or right heart failure); enlargement of the gallbladder (long axis diameter > 8 cm, short-axis diameter > 4 cm); gallstone is incarcerated, debris echo, fluid accumulation around the gallbladder; stripe-like translucent Doppler sign can be observed in the gallbladder wall.

Magnetic resonance imaging: pericholecystic high signal, enlargement of the gallbladder, and gallbladder wall thickening.

Computed tomography: gallbladder wall thickening, fluid accumulation around the gallbladder, enlargement of the gallbladder, and linear high-density areas observed in pericholecystic adipose tissue.

Tc-HIDA scan: normal uptake and excretion of radioactive material, but gallbladder is not visible; Rim sign: radioactivity is increased in gallbladder fossa.

Diagnosis is based on the following: One positive indicator each in A and B; When inflammatory signs are insufficient to diagnose as acute cholecystitis, item C can be used to confirm the diagnosis.

#### Grading Criteria for Acute Cholecystitis^4^

Mild (grade I): meets the requirements for a diagnosis of acute cholecystitis but does not meet the criteria for a diagnosis of moderate or severe grade.

Moderate (grade II): meets any of the following criteria: elevated WBC > 18,000/mm^3^; palpable soft mass in the right upper quadrant; symptoms for >72 hours; local inflammatory markers (bile peritonitis, pericholecystic abscess, liver abscess, gangrenous cholecystitis, and emphysematous cholecystitis).

Severe (grade III): meets any of the following criteria: cardiac insufficiency (hypotension, ≥5 μg/kg min of dopamine or any dose of dobutamine is required to maintain cardiac function), neurological dysfunction (decreased consciousness), respiratory insufficiency (PaO_2_/FiO_2_ ratio < 300), renal insufficiency (oliguria, creatinine > 2.0 mg/dL), hepatic insufficiency (PT-INR > 1.5), and coagulation dysfunction (platelet count < 100,000/mm^3^).

### Criteria for Inclusion and Exclusion

#### Inclusion Criteria

Patients who were hospitalized during emergency treatment in General Surgery Division III of the First Affiliated Hospital of Dalian Medical University between January 2005 and November 2012, met the abovementioned criteria for the diagnosis of acute cholecystitis and underwent laparoscopic surgery or PTGD treatment but did not meet the exclusion criteria were included.

#### Exclusion Criteria

Patients who met any one of the following: chose open cholecystectomy, a history of previous upper abdominal surgery, complication with choledocholithiasis or intrahepatic bile duct stones requiring choledocholithotomy, and complication with other acute abdominal condition requiring surgery, such as appendicitis or intestinal obstruction.

### Treatment Methods

All LC surgeries were performed by surgeons of General Surgery Division III of the First Affiliated Hospital of Dalian Medical University. All surgeries were performed under general anesthesia with the conventional 4-hole antegrade or retrograde cholecystectomy method; if necessary, laparotomy was performed. All PTGD treatments were performed by surgeons in the Intervention Department of the First Affiliated Hospital of Dalian Medical University. Lidocaine (2%) was used as a local anesthetic at the anterior axillary line. A percutaneous transhepatic puncture needle was used to penetrate the gallbladder at the upper 1/3 to withdraw bile. After obtaining a clear cholecystography, a guide wire was inserted and the needle tract was expanded. A 7F pigtail catheter was inserted into the gallbladder to form a loop in the gallbladder, which was connected to an external drainage bag. The bag was sutured and fixed extracorporeally to provide continuous external drainage.

### Emergency LC

After being admitted to the hospital, the patient underwent emergency LC. Intravenous application of second- or third-generation cephalosporins was used for postoperative antiinfection treatment.

### LC After PTGD Treatment

After each patient was admitted to the hospital, physicians from the Intervention Department were immediately invited for a consultation. Under local anesthesia, an emergency PTGD was performed; second- or third-generation of cephalosporins were given at the same time as an antiinfection measure. After the patient's symptoms were alleviated, the patient was discharged. All of the patients returned to the hospital for LC within 1 year.

### LC After Nonsurgical Treatment

After being admitted to the hospital, the patient was not allowed to eat or drink. If necessary, gastrointestinal decompression, intravenous application of second- or third-generation cephalosporins for antiinfection treatment, and intravenous replenishment of volemia were performed. When the patient's condition stabilized, LC treatment was scheduled and performed.

### Only PTGD Treatment

After the emergency patient was admitted to the hospital, physicians from the Intervention Department were immediately invited for a consultation. Under local anesthesia, emergency PTGD treatment was performed with administration of second- or third-generation cephalosporins as an antiinfective. After the patient's symptoms were alleviated, the patient was discharged. These patients did not return to the hospital for LC treatment.

### Evaluation Indicators

We compared the general condition, duration of surgery, intraoperative blood loss, rate of conversion to open surgery, rate of postoperative complications, total fasting time, and total hospitalization time of patients in the first 3 groups. We also compared the efficacy of PTGD treatment with that of nonsurgical treatment. The general conditions and follow-up results of patients who had only PTGD treatment were analyzed.

### Statistical Analysis

All data were analyzed using the professional statistics software SPSS 19.0. Quantitative data are presented as x ± s. The qualitative data were examined using the χ^2^ test, while quantitative data were analyzed by analysis of variance. *P* < 0.05 was considered statistically significant.

## RESULTS

### General Conditions of Patients

Comparison of the general information regarding the laparoscopic surgery patients shows that there is no significant difference in male-to-female ratio or in the incidence of medical illness complications among the 3 groups (*P* > 0.05). The average age of group B patients was 65.6 ± 13.6 years, which is significantly higher than that of both group A patients (59.0 ± 12.9 years) and group C patients (55.4 ± 11.5 years). The proportion of severe acute cholecystitis patients in group B (11.5%) is significantly higher than the proportion in group A (0%) and group C (0%), and the differences are statistically significant (*P* < 0.05). With respect to ASA grading, the proportion of patients in group B with grade III and above cholecystitis (46.2%) is significantly higher than the proportion in group A (21.2%) and group C (14.1%) (*P* < 0.05). By comparing patients’ APACHE II scores, we found that the mean score of the patients in group B (6.0 ± 2.3) was significantly higher than the mean score of patients in group A (4.5 ± 2.4) and group C (3.8 ± 2.1). The mean body temperature of the patients in group B (37.5 ± 0.9 °C) at the time of admission to the hospital was significantly higher than that of the patients in group A (36.8 ± 0.7 °C) and group C (36.5 ± 0.5 °C). The white blood cell counts of patients in group A (12.1 ± 4.3 × 10^3^/mm^3^) and group B (13.6 ± 4.5 × 10^3^/mm^3^) at the time of admission to the hospital were significantly higher than those of group C (8.5 ± 2.5 × 10^3^/mm^3^) (*P* < 0.05) (Table 1).

**TABLE 1:**
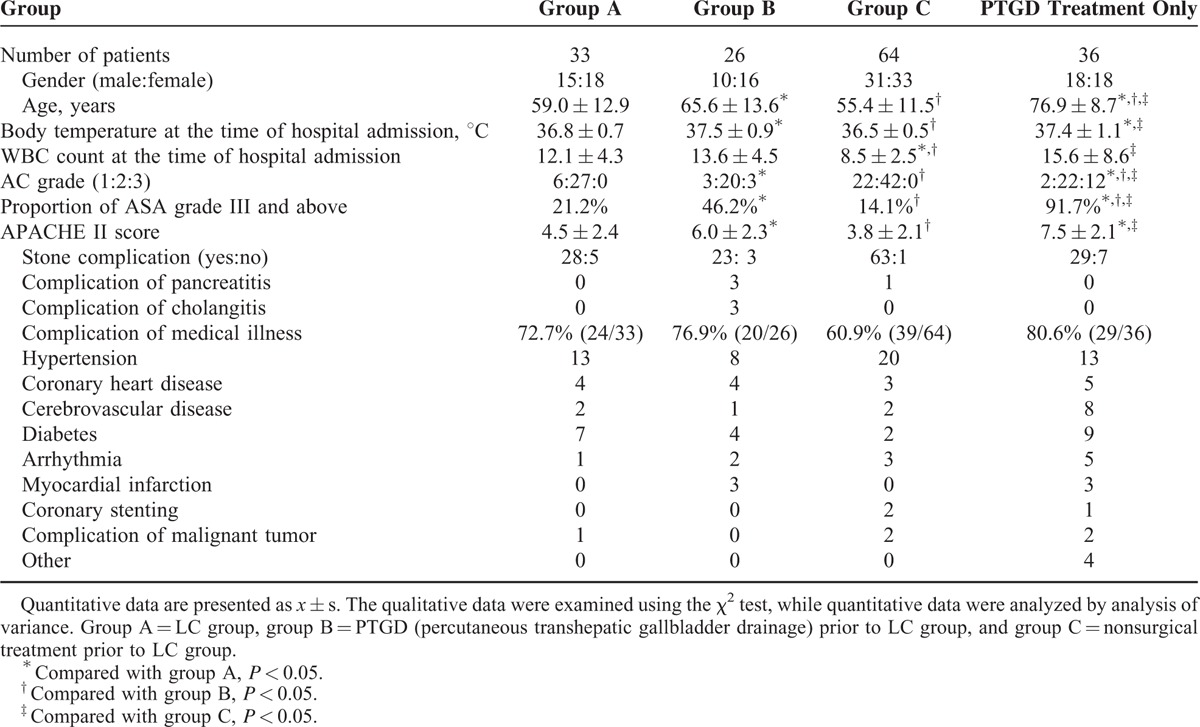
Comparison of Patients’ General Conditions

The “PTGD only” group consisted of 36 patients in total, and their average age was 76.9 ± 8.7 years, significantly higher than the average age of the patients in the aforementioned three groups (*P* < 0.05). Twelve of the patients in this group (33.3%) were severe acute cholecystitis patients, a proportion that is significantly higher than the proportion of severe acute cholecystitis patients in the abovementioned 3 groups (*P* < 0.05). The proportion of patients in the PTGD only group with an ASA grade of III or above was 91.7% (33/36), significantly higher than the proportion in the abovementioned 3 groups (*P* < 0.05). The average APACHE II score was 7.5 ± 2.1, significantly higher than that of the emergency LC group and the group that underwent LC after nonsurgical treatment. However, there is no significant difference from the LC after PTGD treatment group. The average body temperature of the patients in the PTGD only group at the time of admission was 37.4 ± 1.1 °C, significantly higher than that of the patients in the emergency LC group and those in the LC after nonsurgical treatment group (*P* < 0.05); there was no significant difference in mean body temperature from the LC after PTGD treatment group. The average white blood cell count of the PTGD only group at the time of admission was 15.6 ± 8.6 × 10^3^/mm^3^, significantly higher than the average white cell count of the LC after nonsurgical treatment group.

### Comparison of Treatment Efficacy for PTGD Treatment (Group B and PTGD Only Group) and Nonsurgical Treatment

In this study, 62 patients in total underwent PTGD treatment, and 26 of these patients later returned to the hospital for further LC treatment. All patients had successful cannulation without bile leakage, bleeding, or other complications. One patient had drainage tube occlusion 4 weeks later, and another patient's drainage tube fell off 6 weeks later. Both of these patients then returned to the hospital for LC treatment. A third patient had relapse of cholecystitis symptoms 4 weeks later and returned to the hospital. This patient was discharged after the symptoms were alleviated by nonsurgical treatment. There were no hospital deaths. The symptoms of all patients were alleviated by the puncture. Approximately 80.6% (50/62) of the patients had their symptoms alleviated within 24 hours, and the remission rate reached 95.2% (59/62) within 48 hours. The LC surgery after nonsurgical treatment group consisted of 64 patients in total. This group's symptom remission rate within 24 hours after nonsurgical treatment was 15.6% (10/64), and its symptom remission rate within 48 hours was 42.2% (27/64); both of these rates are significantly lower than those of the PTGD treatment group (*P* < 0.05). The average symptom remission time of the PTGD treatment group was 23.6 ± 19.8 hours, significantly shorter than that of the LC after nonsurgical treatment group (72.0 ± 41.0 hours); the difference is statistically significant. Of the patients in the LC after nonsurgical treatment group, 6.3% (4/64) showed no significant remission after an average of 3.5 days of treatment and were then treated with LC. Three of the patients in this group showed pathological changes in the gallbladder, including gangrene, after the operation. The nonremission rate of this group was also significantly higher than that of the PTGD-treated patients (Table 2).

**TABLE 2:**
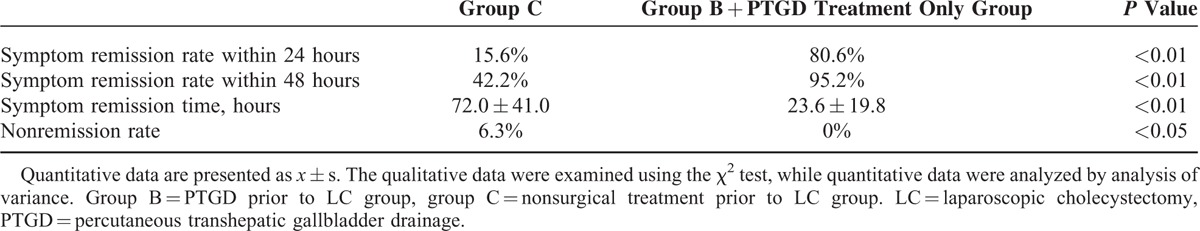
Comparison of Symptom Remission Time in Group B, in the PTGD Treatment Only Group and in the LC After Nonsurgical Treatment Group

### Follow-Up Results of Patients Who Received PTGD Treatment Only

Only 16 families of patients who received PTGD accepted follow-up. Twelve of these patients experienced complications with gallstones, and 4 had further laparotomy treatment in their local hospitals due to relapse of cholecystitis symptoms. The 8 remaining patients had long-term indwelling catheters. The 4 acalculous cholecystitis patients had their catheters removed 2 to 6 months after the puncture. Two patients had relapse of cholecystitis symptoms due to inappropriate eating. They returned to hospital, and their symptoms were alleviated after nonsurgical treatment. The other 2 patients no longer showed cholecystitis symptoms; their average time without a tube was 12 months (6 and 18 months) (Table 3).

**TABLE 3:**
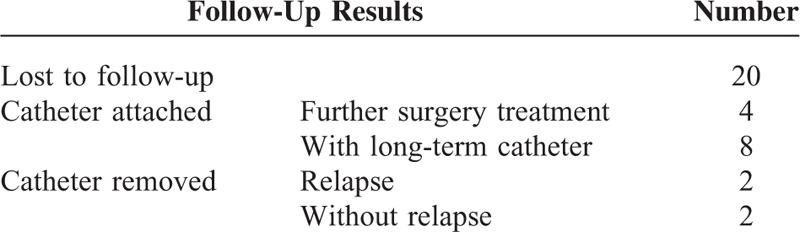
Follow-Up Results of Patients Who Received Percutaneous Transhepatic Gallbladder Drainage (PTGD) Treatment Only

### Comparison of Surgery Results of Emergency LC, LC After PTGD, and LC After Nonsurgical Treatment

This study included a total of 123 LC-treated patients. Eight of them (1 in group A, 5 in group B, and 2 in group C) were converted to laparotomy due to the presence of dense adhesions at the triangle of the gallbladder that could not be separated; the conversion rate was 6.5%. Only 1 patient had postoperative bleeding; in this patient, a second surgery was performed to stop the bleeding after the intervention therapy failed. The conversion rate of patients in group B was 19.2%, significantly higher than that of group A (3.0%), and group C (3.3%) (*P* < 0.05); the difference between group A and group C was not statistically significant. The patients in the 3 groups did not display significant differences in operation time, operation blood loss, or postoperative complication rate (*P* > 0.05). The total hospitalization time of the patients in group B (18.5 ± 4.5 days) was significantly longer than that of the patients in group A (8.2 ± 3.9 days) and group C (10.5 ± 6.4 days) (*P* < 0.05); there was no significant difference between groups A and C. The total fasting time of patients in group B (4.1 ± 1.7 days) and group C (3.4 ± 2.7 days) was significantly longer than that of patients in group A (2.4 ± 1.2 days) (*P* < 0.05); the difference between group B and group C was not statistically significant (Table 4).

**TABLE 4:**
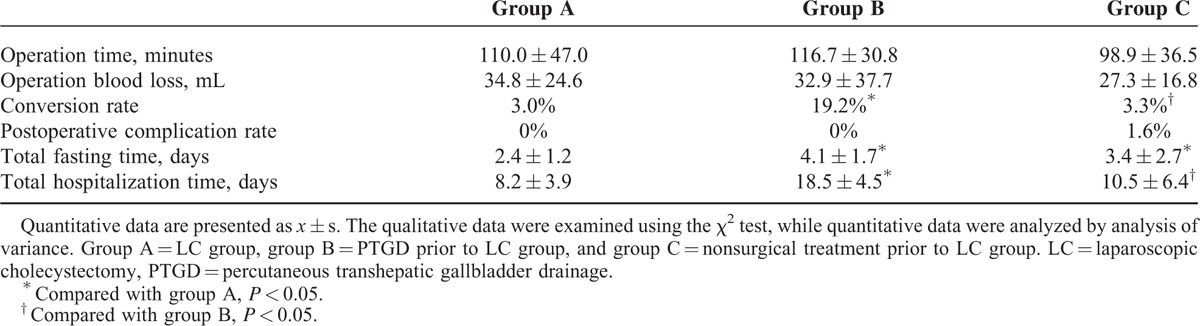
Comparison of Surgery Results for the 3 Groups

On compilation of postoperative pathology of the patients in the 3 groups (Table 5), 7 patients were found to have acute gangrenous cholecystitis (3 in group A and 4 in group C), accounting for 5.7% of all surgery patients (7/123). The difference in the proportion of gangrenous cholecystitis in all 3 groups is not statistically significant (*P* > 0.05). Through comparison of the incidence of acute and chronic inflammation in patients in the 3 groups, we found that the incidence of acute inflammation in patients in group A (60.6%) was significantly higher than in group C (21.8%) (*P* < 0.05); however, the difference compared with group B (42.3%) is not statistically significant.

**TABLE 5:**
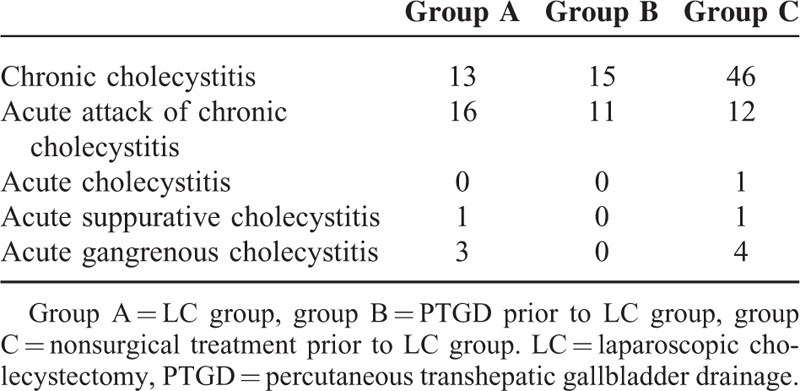
Postoperative Pathology of Laparoscopic Surgery Patients

## DISCUSSION

As laparoscopic techniques are gradually refined, the surgical success rate for acute cholecystitis is gradually increasing. A recent report^5^ showed that its operation time and complication rate already showed no significant difference compared with laparotomy. The prospective study of Kiviluoto et al^6^ even showed that in the treatment of acute cholecystitis patients, the complication rate and postoperative recovery time of LC is lower than OC. Therefore, LC is gradually becoming the preferred treatment for acute cholecystitis. In the present study, 123 acute cholecystitis patients, of whom patients with ASA grade III and above accounted for 22.7% (28/123), underwent LC treatment. The average operation time for all 123 patients was 105.7 ± 38.9 minutes, and the average intraoperative blood loss was 30.5 ± 24.6 mL, similar to literature reports.^7^ Of these patients, 8 had extensive adhesions at the triangle region, and the unclear dissection at the triangle of the gallbladder led to conversion to laparotomy at a rate of 6.5%. One patient had postoperative bile leakage and biliary tract bleeding. After the interventional treatment failed to improve this condition, a second operation was performed to stop the bleeding. No other patients had obvious postoperative complications. The incidence rate for complications was 0.8%, and there was no hospital death. The present study data show that LC is safe and effective for the treatment of acute cholecystitis. Indeed, it has gradually become the preferred treatment for acute cholecystitis patients, while an opportune conversion to laparotomy can reduce the incidence of postoperative complications and mortality.

In 1980, Radder^3^ reported the first case of percutaneous transhepatic gallbladder drainage in the treatment of suppurative cholecystitis. The basic principle of this procedure is the reduction of gallbladder tension by drainage of bile through a gallbladder drainage catheter. In combination with intravenous treatment to prevent infection, acute cholecystitis symptoms can be alleviated by this method, thus making it possible to defer surgical treatment. With progress in this field, the percutaneous transhepatic gallbladder drainage method and its treatment efficacy have been increasingly accepted by researchers. Studies from Borzellino et al^8^ showed that PTGD can reduce the mortality of acute cholecystitis patients, whereas Melloul et al,^9^ reviewing 14 reports on percutaneous gallbladder drainage published abroad between 1998 and 2010, found that the success rate of puncture was close to 100%. The symptom remission rate reached 78% to 100%, whereas the complication rate was only 3% to 13% and the mortality was 0% to 11%. These findings confirm that percutaneous transhepatic gallbladder drainage is safe and effective in the treatment of high-risk acute cholecystitis patients.

In the present study, 62 patients in total underwent PTGD treatment. All patients received puncture treatment in the Intervention Department under B-mode ultrasound guidance, with a success rate of 100%. Later, 41.9% (26/62) of these patients had further LC; 58.1% (36/62) of these patients were discharged from the hospital with catheters, and they did not return to the hospital for cholecystectomy. One of the patients had drainage tube occlusion 4 weeks later, and another patient's drainage tube fell off 6 weeks later. Both patients then returned to hospital for LC treatment. A third patient had fever, nausea, and vomiting again 4 weeks after drainage. This patient was treated according to the symptoms and was discharged after the symptoms had been alleviated. No bile leakage, bleeding, or other complications occurred in any of the patients. The symptom remission rate within 24 hours of puncture was 80.6% (50/62), and the remission rate reached 95.2% (59/62) within 48 hours; both of these rates are significantly higher than the rates achieved in the group of patients who underwent LC after nonsurgical treatment. The average symptom remission time (23.6 ± 19.8 hours) was also significantly shorter than in the patients who underwent LC after nonsurgical treatment, for whom it was 72.0 ± 41.0 hours. Based on these findings, we conclude that PTGD can alleviate the symptoms of acute cholecystitis patients, especially those in whom symptoms are severe, in a short period of time, that it has lower complication rate, and that its efficacy is certain and safe.

However, PTGD is merely a transition method, and most acute cholecystitis patients later require further cholecystectomy. Whether PTGD has any impact on the difficulty of later surgery has been an issue for study. Comparing LC after PTGD treatment patients to emergency LC patients, a retrospective study by In-Gyu et al^10^ showed that patients in the LC after PTGD treatment group had better results with respect to operation time, and the difference in the rate of conversion to laparotomy compared with emergency LC group patients was statistically significant. However, the total hospitalization time of the patients in the emergency LC group was significantly shorter than that of the patients in the LC after PTGD treatment group (*P* < 0.01). Kim et al^11^ found that, compared with surgery group patients without PTGD treatment, patients who underwent surgery after PTGD treatment had longer operation times, whereas the study of Tsumura et al^12^ showed no significant difference in surgery results with or without PTGD treatment. A report from another research group^13^ showed that PTGD not only did not shorten the operation time for the deferred surgery or the postoperative hospitalization time but also increased the rate of intraoperative conversion to laparotomy. The data from the present study show that patients in the LC after PTGD, emergency LC, and LC after nonsurgical treatment groups experienced no significant differences in operation time, intraoperative blood loss, or postoperative complication rate. However, the operation conversion rate of the LC after PTGD group patients was significantly higher than that of the emergency LC and LC after nonsurgical treatment groups (19.2% vs 3.0% vs 3.3%, respectively), and the differences were statistically significant. By comparing the general conditions of the patients in the 3 groups, we observed that the proportion of severe acute cholecystitis patients, the proportion of ASA grade III and above patients and the APACHE II scores of the LC after PTGD group patients were all significantly higher than those of the patients in the other 2 groups. In addition, patients in this group had worse situations with respect to disease severity and general condition than patients in groups A and C. The higher operation conversion rate of the patients in group B might be related to these factors. The fact that the LC after PTGD group patients had longer hospitalization times than the patients in the other 2 groups may be because PTGD patients needed to be hospitalized twice to complete their treatment and that the general conditions of the LC after PTGD group patients were worse than those of the patients in the other 2 groups. The preoperative evaluation for patients in this group is also more detailed than for the patients in the other 2 groups; therefore, the hospitalization time is longer than for patients in the emergency LC and LC after nonsurgical treatment groups.

There are many reports in the literature on whether acute cholecystitis should be treated with emergency LC. Knight et al^14^ reported that there was no significant difference in the rate of conversion to laparotomy when the LC was performed within 3 days or after 3 days of disease onset. Tzovaras et al^15^ confirmed this finding. In contrast, Bender and Zenilman^16^ emphasized that emergency surgery is better for acute cholecystitis patients. Koo and Thirbly^17^ also believed that the surgery should not be delayed for acute cholecystitis patients. Studies from Madan et al^18^ also showed that acute cholecystitis patients who underwent surgery within 48 hours had shorter operation times, hospitalization times, and postoperative hospitalization times than patients for whom surgery was deferred, and the difference was statistically significant. In the present study, we compared the emergency LC group patients with LC after nonsurgical treatment group patients and found that there is no significant difference in operation time, rate of intraoperative conversion to laparotomy, or postoperative complication rate. However, the total food fasting time of emergency surgery patients is shorter than that of patients who underwent operation after nonsurgical treatment (*P* < 0.05). Thus, although there is no significant difference in the surgery results for emergency surgery and LC after nonsurgical treatment for acute cholecystitis patients, emergency surgery can shorten food fasting time and result in a better quality of life for patients.

For high-risk elderly acute acalculous cholecystitis patients, we need to consider not only the thoroughness of treatment but also its safety. Comparing PTGD treatment with emergency laparotomy surgery for these patients, domestic researchers^19^ found that the symptoms and signs of 13 acalculous cholecystitis patients in PTGD group disappeared completely and that they were cured without further surgery. Through an average of 32-month follow-up, Chung et al^20^ found that only 7.14% (2/28) of acalculous cholecystitis patients who received only PTGD treatment had relapse of symptoms; all the other patients were cured by the initial treatment. Sugiyama et al^21^ also reported 12 cases of acalculous cholecystitis patients who underwent percutaneous cholecystostomy and in whom no relapse was found after extubation with an average follow-up of 1.7 years. The follow-up data in the present study showed that acute cholecystitis symptoms and signs did not return in 2 acalculous cholecystitis patients in whom the catheter was removed 3 months after PTGD treatment and that the average time of these patients without a tube was 12 months. The other 2 acalculous cholecystitis patients had relapse of cholecystitis due to inappropriate eating after extubation. Their symptoms were alleviated after nonsurgical treatment. LC can relieve the inflammatory response rapidly in most patients. However, LC can lead to high morbidity and mortality in most elderly acute cholecystitis patients. Especially in elderly ones with complex cholecystitis, LC can lead to higher rates of conversion to open cholecystectomy, as well as increased postoperative complications and longer lengths of hospital stay. When performing LC, we should avoid the most severe complication, biliary injury. PTGD is performed as a simple operation, without high requirement for sophisticated equipment, with lesser trauma and quick recovery, and is highly economical.^22^ However, the risks, such as bowel injury, bile leakage, pneumothorax, or subhepatic hematoma, should be avoided. In summary, for suitable elderly acalculous cholecystitis patients, PTGD may be able to cure the patients in the initial treatment. However, due to limited acquisition of follow-up data in the present study, we cannot determine the initial treatment effect of PTGD on acute acalculous cholecystitis from the present data. A multicenter large sample randomized control study is needed before we can reach a conclusion.

## CONCLUSION

When there are emergency surgery contraindications, PTGD may be safe and effective for the treatment of high-risk elderly patients, and it can alleviate patients’ symptoms within a short period of time. For acute cholecystitis patients without surgery contraindications, emergency surgery should be performed as soon as possible after diagnosis. The present study will benefit the diagnosis and treatment of high-risk elderly acute cholecystitis patients. Therefore, we recommend the readers to apply PTGD into routine clinical practice.

### Uncited reference

^10^
